# Obesidade e interseccionalidade: análise crítica de narrativas no âmbito das políticas públicas de saúde no Brasil (2004-2021) 

**DOI:** 10.1590/0102-311XPT240322

**Published:** 2023-07-17

**Authors:** Lorrany Santos Rodrigues, Nayara Garcez Miranda, Danielle Cabrini

**Affiliations:** 1 Escola Superior de Ciências da Saúde, Universidade de Brasília, Brasília, Brasil.; 2 Secretaria de Estado de Saúde do Distrito Federal, Brasília, Brasil.; 3 Universidade Federal do Espírito Santo, Vitória, Brasil.

**Keywords:** Análise de Gênero na Saúde, Enquadramento Interseccional, Obesidade, Programas e Políticas de Nutrição e Alimentação, Atenção Primária à Saúde, Gender Analysis in Health, Intersectional Framework, Obesity, Nutrition Programs and Policies, Primary Health Care, Analisis de Género en la Salud, Marco Interseccional, Obesidad, Programas y Políticas de Nutrición y Alimentación, Atención Primaria de Salud

## Abstract

Objetivou-se realizar uma análise crítica da narrativa das políticas públicas de saúde brasileiras no cuidado da obesidade a partir de uma perspectiva interseccional. Trata-se de estudo qualitativo exploratório, documental e analítico, baseado na abordagem “What’s the problem represented to be?” *[“Qual é o problema representado para ser?”]*, conhecida como WPR. Tal abordagem se configura como uma ferramenta metodológica de análise crítica de políticas públicas a partir de seis perguntas norteadoras. Foram selecionados dez documentos, publicados entre 2004 a 2021 pelo governo brasileiro. A análise crítica resultou em três categorias: (i) causas da obesidade e narrativa dominante: quais são os problemas representados?; (ii) narrativa dominante e cuidado em saúde: quais são os efeitos para as pessoas com obesidade?; e (iii) obesidade e interseccionalidade: onde estão os silêncios? O consumo de alimentos e o sedentarismo foram a narrativa dominante como causas da obesidade. A interseccionalidade, mediada pelas categorias de gênero/sexo, raça/cor e classe social, foi identificada como um silêncio na narrativa das políticas públicas de saúde. Tais categorias não foram consideradas como causas atreladas à obesidade, tampouco foram incluídas de forma efetiva nas ações propostas pelas políticas públicas de saúde. Os silêncios encontrados no estudo destacam a necessidade de inclusão da interseccionalidade na elaboração e execução de políticas públicas de saúde e no cuidado das pessoas com obesidade. Tendo em vista as intersecções de gênero/sexo, raça/cor e classe social e suas formas de opressão com o surgimento e agravo da obesidade, são de extrema relevância análises críticas sobre as narrativas simplistas nas políticas públicas de saúde para problematização das lacunas que repercutem no cuidado dos usuários com obesidade.

## Abstract (en)

This study aimed to critically analyze the narrative of Brazilian public health policies in obesity care based on an intersectional approach. This is a qualitative exploratory, documentary, and analytical study based on the “What’s the problem represented to be?” approach (WPR). This approach constitutes a methodological instrument for critical analysis of public policies based on six guiding questions. A total of ten documents were selected, published from 2004 to 2021 by the Brazilian government. The critical analysis resulted in three categories: (i) obesity causes and the dominant narrative: what problems are represented?; (ii) dominant narrative and health care: what are the effects for people with obesity?; (iii) obesity and intersectionality: where are silences? The consumption of food and sedentary lifestyle were the dominant narrative as causes of obesity. Intersectionality, mediated by the categories of gender/sex, race/skin-color, and social class, was identified as silenced in the narrative of public health policies, not being associated as linked causes of obesity, nor effectively included in the proposed actions of the policies. The silences found in the study highlight the need to include intersectionality in the elaboration and execution of public health policies and in the care of people with obesity. Considering the intersections of gender/sex, race/skin-color, and social class and their forms of oppression in the emergence and aggravation of obesity, critical analyses of simplistic narratives in public health policies are extremely relevant to problematize gaps affect the care of users with obesity.

## Resumen

Este estudio tuvo como objetivo realizar un análisis crítico de la narrativa de las políticas públicas de salud brasileñas en el cuidado de la obesidad con base en un enfoque interseccional. Estudio cualitativo exploratorio, documental y analítico. Basado en el enfoque “*Whats the problem represent to be?*” [¿Cuál es el problema representado?], conocido como WPR. Tal enfoque se configura como una herramienta metodológica para el análisis crítico de las políticas públicas con base en seis preguntas rectoras. Se seleccionaron 10 documentos, publicados entre el 2004 y el 2021 por el gobierno brasileño. El análisis crítico resultó en tres categorías: (i) causas de la obesidad y la narrativa dominante: ¿Qué problemas se representan?; (ii) narrativa dominante y el cuidado en salud ¿Cuáles son los efectos para las personas con obesidad?; (iii) obesidad e interseccionalidad ¿Dónde están los silencios?. El consumo de alimentos y el sedentarismo fueron la narrativa dominante como causas de la obesidad. La interseccionalidad, mediada por las categorías de género/sexo, raza/color y clase social fue identificada como un silencio en la narrativa de las PPS, sin asociarlas como causas vinculadas a la obesidad ni incluirlas de forma efectiva en las acciones propuestas por las políticas públicas de salud. Los silencios encontrados en el estudio resaltan la necesidad de incluir la interseccionalidad en la elaboración y ejecución de las políticas públicas de salud y en el cuidado de las personas con obesidad. Considerando las intersecciones de género/sexo, raza/color y clase social y sus formas de opresión con el surgimiento y agravamiento de la obesidad, es sumamente relevante realizar análisis críticos sobre las narrativas simplistas en las políticas públicas de salud, para problematizar las brechas que repercuten en el cuidado de los usuarios con obesidad.

## Introdução

A obesidade tem sido retratada como problema de saúde pública e fator de risco para o surgimento e/ou agravamento de diversas doenças crônicas não transmissíveis (DCNT) ^1^^,^^2^. Suas causas são multidimensionais e demonstram interdependência entre fatores biológicos, sociais, econômicos, políticos, culturais e ambientais, o que permite compreender a obesidade sob uma ótica de sindemia ^3^^,^^4^^,^^5^.

Na América Latina, a taxa de obesidade entre mulheres é maior do que entre homens ^4^^,^^6^^,^^7^^,^^8^. No Brasil, a prevalência de adultos com obesidade é de 22,4%, sendo mais expressiva na população com baixa escolaridade e renda ^9^, além de figurar como o terceiro fator de risco responsável por incapacidades e mortes em mulheres, ocupando o quinto lugar entre os homens ^10^.

Ao considerar os aspectos raciais, de gênero/sexo e a classe social, a obesidade impacta os indivíduos de formas distintas, principalmente nos países em desenvolvimento, onde há profundas desigualdades e iniquidades sociais, como o Brasil ^11^^,^^12^^,^^13^^,^^14^^,^^15^^,^^16^. O gênero/sexo, a raça/cor e a classe social são aspectos intrínsecos ao processo de construção dos indivíduos. Dessa forma, esses marcadores sociais requerem compreensão interseccional ^17^^,^^18^^,^^19^^,^^20^^,^^21^^,^^22^^,^^23^^,^^24^.

Para Collins & Bilge ^25^ (p. 15) “*a interseccionalidade investiga como as relações interseccionais de poder influenciam as relações sociais em sociedades marcadas pela diversidade, bem como as experiências individuais da vida cotidiana. Como ferramenta analítica, a interseccionalidade considera que as categorias de raça, classe, gênero, orientação sexual, nacionalidade, capacidade, etnia e faixa etária - entre outras - são inter-relacionadas e moldam-se mutuamente. A interseccionalidade é uma forma de entender e explicar a complexidade do mundo, das pessoas e das experiências humanas*”. Para as autoras, o uso da interseccionalidade como ferramenta analítica evoca seis ideias centrais: a desigualdade social, as relações de poder interseccionais, o contexto social, a relacionalidade, a justiça social e a complexidade.

A partir de uma análise das inter-relações entre gênero/sexo, raça/cor e obesidade, o estudo de Ferreira et al. ^26^ identificou que mulheres negras apresentaram maiores chances de serem classificadas com obesidade quando comparadas a mulheres brancas. Do mesmo modo, Silva & Cabrini ^11^ conduziram um estudo que traçou o perfil biográfico da obesidade em mulheres brasileiras beneficiárias do programa de transferência de renda Bolsa Família, no qual 71,6% das mulheres com obesidade participantes do estudo eram negras e 50% eram as únicas responsáveis pela renda da família.

Tais achados revelam a correlação entre os marcadores sociais apresentados e a maior vulnerabilidade à obesidade e reforçam a centralidade das políticas públicas de saúde nessa agenda como medidas de intervenção nos problemas apontados. Entretanto, políticas públicas de saúde construídas sem considerar os determinantes sociais da saúde tendem a perpetuar uma série de desconexões com a realidade vivida pelas pessoas que pretendem alcançar ^27^^,^^28^^,^^29^. Tendo em vista o cenário exposto, a incorporação da interseccionalidade na problematização e análise das políticas públicas de saúde voltadas ao cuidado das pessoas com obesidade é um caminho. Logo, o objetivo do estudo foi realizar uma análise crítica da narrativa das políticas públicas de saúde brasileiras no cuidado das pessoas com obesidade a partir de uma abordagem interseccional.

## Métodos

### Desenho do estudo

Trata-se de um estudo qualitativo exploratório, documental e analítico, baseado na abordagem “*What’s the problem represented to be?*” [“Qual é o problema representado para ser?”], conhecida como WPR ^27^. Tal abordagem é uma ferramenta metodológica de análise crítica de políticas públicas a partir de perguntas norteadoras ^27^^,^^30^^,^^31^ e sustenta a ideia de que políticas públicas não são apenas reações a problemas estáticos. A partir da WPR, é possível observar que as ações apresentadas nas políticas públicas dizem implicitamente o que é considerado problema e o que precisa ser combatido e/ou modificado, traduzindo, desse modo, quais são os problemas representados por cada políticas públicas ^27^^,^^30^^,^^31^.

A abordagem WPR convida para uma mudança de foco na análise de políticas públicas, na medida em que se concentra em analisar criticamente como os problemas estão representados e como eles são abordados nas políticas públicas ^27^^,^^30^^,^^32^. A WPR possibilita compreender os possíveis efeitos das políticas públicas em seu público-alvo e as lacunas no processo de formulação e implementação, além de questionar como são construídas as narrativas dominantes e seus processos de legitimação. Ela auxilia, por fim, na identificação de quais pontos necessitam ser revistos, questionados e/ou substituídos nas políticas públicas.

Para conduzir uma análise crítica de narrativa, a abordagem WPR apresenta um conjunto de seis questões facilitadoras (Quadro 1) ^27^^,^^28^^,^^32^^,^^33^. Com vistas a contemplar o objetivo deste estudo, sem alterar, portanto, o sentido das questões da WPR, as autoras realizaram adaptações e tradução das questões facilitadoras para a língua portuguesa. Vale ressaltar que as pesquisadoras têm participação ativa no emprego da abordagem WPR e são consideradas parte da representação do problema analisado, a partir da problematização e reflexão durante todo o processo de análise crítica ^27^. Entendendo sua inserção metodológica, a WPR, bem como esta pesquisa, não pretende realizar avaliação de eficácia e eficiência das políticas públicas, mas sim uma análise crítica de narrativas dos problemas representados nas políticas públicas de saúde ^28^. O estudo foi realizado entre agosto de 2020 e janeiro de 2022.

**Quadro 1:** Questões “*What’s the problem represented to be?*” [“Qual é o problema representado para ser?”].

QUESTÃO	
1	Qual é o “problema” representado em uma política ou proposta de política específica? (Qual o problema implícito?)
2	Quais pressupostos ou suposições sustentam essa representação do “problema”? (O que sustenta esse problema?)
3	Como surgiu essa representação do “problema”? (Como se tornou legítimo?)
4	O que não é problematizado nesta representação do “problema”? Onde estão os silêncios? O "problema" pode ser pensado de maneira diferente? (Quais os silêncios? O que é omitido?)
5	Quais efeitos são produzidos por essa representação do “problema”? (Qual o efeito nas pessoas com obesidade e no cuidado?)
6	Como/onde essa representação do 'problema' foi produzida, disseminada e defendida? Como foi (ou poderia ser) questionada, interrompida e substituída? (Como se tornou dominante? E como questioná-las?)

Fonte: Bacchi ^27^^,^^33^, tradução das autoras.

### Critérios de elegibilidade

Foram incluídos no estudo políticas públicas de saúde e documentos oficiais que abordam a promoção à saúde e a prevenção da obesidade, com centralidade na atenção primária à saúde (APS), disponíveis em *sites* oficiais e na biblioteca virtual do Ministério da Saúde. Um recorte temporal de vinte anos foi utilizado, considerando o intervalo de 2001 a 2021. Para facilitar o entendimento, optou-se no decorrer do estudo pela utilização do termo “documentos”, em referência aos documentos e às políticas públicas de saúde analisados.

Os documentos que contemplavam os níveis de atenção à saúde secundário e/ou terciário, sobre cuidados com a obesidade infantil e o tratamento da obesidade, foram inelegíveis ao estudo. Para a pesquisa dos documentos, foram utilizadas as palavras-chave: “obesidade”, “manejo da obesidade”, “atenção primária à saúde”, “programas e políticas de alimentação e nutrição” e “prevenção da obesidade” - isoladas e combinadas entre si.

### Análise de dados

Após a seleção dos documentos, a análise crítica foi realizada em três etapas. Na primeira, foi feita a leitura na íntegra dos documentos selecionados em busca de informações-chave sobre os marcadores sociais gênero/sexo, raça/cor e classe social, orientada pela abordagem teórico-metodológica da interseccionalidade ^18^^,^^20^^,^^22^^,^^34^. Os achados foram compilados em planilha no programa Microsoft Excel (https://products.office.com/).

A segunda etapa consistiu em responder às perguntas da WPR para cada documento analisado (Quadro 1), com base nas informações extraídas da etapa anterior, e compilar as respostas em nova planilha do programa Microsoft Excel. A criação de categorias de análise foi a última etapa e é fruto da compilação das respostas das seis questões contidas na WPR, fundamentada no estudo realizado por Salas et al. ^28^ e na abordagem teórico-metodológica da interseccionalidade ^17^^,^^18^^,^^19^^,^^20^^,^^21^^,^^22^^,^^23^. Os marcadores sociais de gênero/sexo foram combinados com vistas a contemplar as múltiplas expressões biológicas e de construção social. Suas dimensões foram observadas na análise conforme descreve Butler ^34^. Para raça/cor, o estudo adotou o recorte de pessoas negras (pretas e pardas) ^17^, enquanto para classes sociais, foram consideradas as de menor renda ^17^. Essa escolha se deu a partir das particularidades que envolvem as iniquidades em saúde e de um primeiro olhar sob a perspectiva de análise de narrativas das políticas públicas, expressando o que considerou-se como a “primeira camada” das relações de poder imbricadas no processo.

### Aspectos éticos e legais da pesquisa

O estudo foi realizado com documentos de acesso público, portanto, isento de aprovação em Comitê de Ética em Pesquisa. Foram assegurados os preceitos éticos legais estabelecidos pela *Resolução nº 510*, de 7 de abril de 2016, do Conselho Nacional de Saúde do Brasil.

## Resultados

Foram selecionados dez documentos, publicados entre 2004 até 2021 (Quadro 2). A análise crítica de narrativa a partir das questões WPR resultou em três categorias: (i) causas da obesidade e narrativa dominante: quais são os problemas representados?; (ii) narrativa dominante e cuidado em saúde: quais são os efeitos para as pessoas com obesidade?; e (iii) obesidade e interseccionalidade: onde estão os silêncios? (Quadro 3).

**Quadro 2:** Documentos e políticas públicas de saúde selecionadas para análise no estudo.

DOCUMENTOS ANALISADOS	ANO
Política Nacional de Atenção Integral à Saúde da Mulher (PNAISM) ^38^	2004
Política Nacional de Segurança Alimentar e Nutricional (PNSAN) ^40^	2010
Política Nacional de Alimentação e Nutrição (PNAN) ^5^	2012
Política Nacional de Promoção da Saúde (PNPS) ^29^	2014
*Cadernos de Atenção Básica nº 38*^35^	2014
*Cadernos de Atenção Básica nº 35*^36^	2014
*Guia Alimentar para a População Brasileira*^42^	2014
*Estratégia Intersetorial de Prevenção e Controle da Obesidade: Recomendação para Estados e Municípios*^35^	2014
*Perspectivas e Desafios no Cuidado às Pessoas com Obesidade no SUS: Resultados do Laboratório de Inovação no Manejo da Obesidade nas Redes de Atenção à Saúde*^39^	2014
Plano de Ações Estratégicas para o Enfrentamento das Doenças Crônicas Não Transmissíveis (DCNT) no Brasil 2011-2022 ^41^	2021

**Quadro 3:** Problemas representados e soluções propostas nos documentos e políticas públicas de saúde analisadas.

DOCUMENTOS ANALISADOS	CAUSAS DA OBESIDADE E A NARRATIVA DOMINANTE: QUAIS OS PROBLEMAS REPRESENTADOS?	NARRATIVA DOMINANTE E O CUIDADO EM SAÚDE: QUAIS OS EFEITOS PARA AS PESSOAS COM OBESIDADE?	OBESIDADE E INTERSECCIONALIDADE: ONDE ESTÃO OS SILÊNCIOS?
Política Nacional de Atenção Integral à Saúde da Mulher (PNAISM) ^38^	Alimentação inadequada e sedentarismo. Adoecimento feminino mais associado a questões sociais do que biológicas.	A narrativa é integral, mas o cuidado focado na reprodução cria um paradoxo. Reforço da saúde atrelado ao sistema reprodutor.	As intervenções são atreladas a doenças crônicas não transmissíveis (DCNT), reprodução/climatério. Silêncios sobre o cuidado às mulheres com obesidade e a relação com raça/cor, classe social e gênero na integralidade em saúde.
Política Nacional de Segurança Alimentar e Nutricional (PNSAN) ^40^	Produção, acesso e consumo de alimentos. Renda e vulnerabilidade social. Desconhecimento sobre alimentação adequada e saudável.	Relação entre insegurança alimentar e nutricional sem contextualizar os determinantes coletivos, tende a reforçar a centralidade nas ações individuais.	A obesidade não é citada. Vigilância alimentar e nutricional pouco direcionada a abordagem das vulnerabilidades sociais e organização de cuidado equitativo.
Plano de Ações Estratégicas para o Enfrentamento das Doenças Crônicas Não Transmissíveis (DCNT) no Brasil 2011-2022 ^41^	Alimentação inadequada e sedentarismo. Pobreza e vulnerabilidade social, com foco na renda. Desconhecimento sobre alimentação adequada e saudável.	Centralidade no indivíduo, e no conhecimento para escolhas alimentares, reduz a complexidade do fenômeno. Cuidado individual.	Gênero e raça/cor não são abordados, tampouco incluídos nas ações. Silêncios acerca dos determinantes das escolhas alimentares.
Política Nacional de Alimentação e Nutrição (PNAN) ^5^	Alimentação inadequada e sedentarismo. A renda e a determinação social afetam a obesidade.	Responsabilização dos usuários, a partir do foco nas escolhas alimentares e atividade. Induz cuidado centrado no balanço energético.	A verticalidade das causas atribuídas à obesidade cria silêncios associados a questões de raça/cor, classe e gênero, reforçando a responsabilidade individual.
Política Nacional de Promoção da Saúde (PNPS) ^29^	Determinação social e vulnerabilidade. Pobreza e a garantia da segurança alimentar e nutricional e direito humano à alimentação adequada (DHAA).	O foco na redução da pobreza para garantia de segurança alimentar e nutricional e DHAA atribui à renda *status* de solução, desconexa de uma discussão interseccional. Indutora de um cuidado setorizado.	Silêncios sobre relação direta dos demais determinantes sociais da sáude (DSS), como raça/cor e gênero para alimentação adequada e saudável. Foco na renda e pobreza associado ao acesso e consumo.
*Cadernos de Atenção Básica nº 38*^37^	Alimentação inadequada e sedentarismo. Comportamentos disfuncionais.	Simplificação das questões relacionadas à obesidade e solução mediante correção/ajuste de comportamentos.	Silêncios sobre a complexidade da obesidade, bem como raça/cor e classe social na adoção de ações equitativas. Silêncio entre a relação de gênero e obesidade para além da reprodução.
*Cadernos de Atenção Básica nº 35*^36^	Alimentação inadequada e sedentarismo. Relação com os determinantes sociais de saúde.	Hierarquia do manejo clínico biológico sobre os aspectos sociais, reforçam que o cuidado da obesidade reside no ajuste de comportamentos disfuncionais.	Raça/cor, gênero e classe social são silêncios percebidos no documento. Foco nos marcadores biológicos de saúde. Orientações não alinhadas com o *Guia Alimentar para a População Brasileira*.
*Guia Alimentar para a População Brasileira*^42^	Consumo de alimentos ultraprocessados e composição nutricional desbalanceada. Balanço energético positivo. Conhecimento sobre alimentação adequada e saudável.	Foco nas escolhas alimentares, descontextualizada dos determinantes da alimentação, tende a incidir nas pessoas com obesidade e no cuidado ofertado, reforçando a responsabilidade individual, e a culpabilização por comportamentos divergentes à orientação.	Silêncios sobre determinantes da alimentação. Estratégias focadas na escolha dos alimentos. Orientações igualitárias, mas não equânimes.
*Estratégia Intersetorial de Prevenção e Controle da Obesidade: Recomendação para Estados e Municípios*^35^	Alimentação inadequada e sedentarismo. Ambientes interferem no consumo de alimentos e atividade física.	Discurso intersetorial, mas o foco em ações específicas pode atuar na construção de um discurso amplo, mas divergente da prática e das orientações, que ainda são focais na obesidade, e não no indivíduo.	A narrativa intersetorial, mas foco na produção, acesso, consumo de alimentos e na prática de atividade física. Silêncio nas ações, que se configuram como multissetoriais, ao invés de intersetoriais. Silêncios sobre a categoria de gênero, descrita, mas não incluída nas ações.
*Perspectivas e Desafios no Cuidado às Pessoas com Obesidade no SUS: Resultados do Laboratório de Inovação no Manejo da Obesidade nas Redes de Atenção à Saúde*^39^	Obesidade tem causas múltiplas e é um problema social. Determinação social é um problema na obesidade.	A ampliação do debate sobre os determinantes sociais de saúde e da obesidade, contribui para um cuidado integral, e ainda para que ações individuais sejam parte das estratégias, e não o foco.	Gênero e o ganho de peso excessivo na gestação e a paridade ganham destaque, associados à reprodução. Silêncios sobre as inter-relações das categorias na obesidade.

### Causas da obesidade e narrativa dominante: quais são os problemas representados?

Os documentos analisados apresentaram discurso congruente sobre as causas da obesidade. A narrativa dominante, presente na extensa maioria dos documentos analisados, destaca que o balanço energético positivo, ou seja, o consumo excessivo de calorias associado à inexistência da prática de atividade física, é a causa central da obesidade. Como exemplos, destacam-se a *Estratégia Intersetorial de Prevenção e Controle da Obesidade: Recomendação para Estados e Municípios*^35^, os *Cadernos de Atenção Básica* nº 35 ^36^ e nº 38 ^37^ e a Política Nacional de Atenção Integral à Saúde da Mulher (PNAISM) ^38^, em que a narrativa da obesidade como multifatorial surge nas introduções e apresentações de forma breve e conceitual, mas é pouco explorada nos documentos.

A série técnica *Perspectivas e Desafios no Cuidado às Pessoas com Obesidade no SUS: Resultados do Laboratório de Inovação no Manejo da Obesidade nas Redes de Atenção à Saúde*^39^ já apresenta uma narrativa mais ampla. Reforça que a obesidade é um problema social multifatorial e que o balanço energético positivo é resultado de múltiplos fatores, e não a causa em si. Destaca, ainda, que as condições de trabalho, moradia e segurança devem ser incorporadas como determinantes atrelados ao surgimento da obesidade. A Política Nacional de Segurança Alimentar e Nutricional (PNSAN) ^40^, apesar de não abordar diretamente a obesidade, afirma que as causas para a insegurança alimentar e nutricional são amplas e aprofundadas pela vulnerabilidade social. Nesse contexto, importa reforçar a obesidade enquanto uma expressão de insegurança alimentar e nutricional.

Os determinantes sociais da saúde (DSS) e a vulnerabilidade social são apresentados de forma conceitual, e não atrelados às causas da obesidade. Quando citada, a classe social é mediada pela discussão da renda e assume posição de destaque como uma das causas e/ou agravantes da obesidade, a exemplo do descrito na Política Nacional de Alimentação e Nutrição (PNAN) ^5^ e no Plano de Ações Estratégicas para o Enfrentamento das Doenças Crônicas Não Transmissíveis (DCNT) no Brasil 2011-2022 ^41^. A pobreza e a escolaridade são apresentadas como causas relacionadas, ancoradas na compreensão de que a falta de conhecimento implica escolhas alimentares inadequadas e não saudáveis. Esse é um dos problemas representados pelo *Guia Alimentar para a População Brasileira*^42^.

Os DSS são abordados a partir das possibilidades de auxiliar ou dificultar a narrativa dominante, centrada no balanço energético positivo. A classe social, por exemplo, é referida nos documentos a partir do impacto da renda na aquisição de alimentos, mas não é contextualizada aos demais efeitos de tal determinante no cuidado das pessoas com obesidade.

### Narrativa dominante e o cuidado: quais são os efeitos nas pessoas com obesidade e no cuidado ofertado?

O cuidado intersetorial é citado como necessário na atenção à saúde de indivíduos e coletividades com obesidade, mas, embora os documentos apresentem essa discussão conceitual, não foi percebida sua incorporação nas ações, pois elas se concentram nas “soluções” para a narrativa dominante. Surge, assim, uma contradição entre a narrativa da intersetorialidade e as ações propostas, como observado nos *Cadernos de Atenção Básica* nº 35 ^36^ e nº 38 ^37^ e no Plano de Ações Estratégicas para o Enfrentamento das Doenças Crônicas Não Transmissíveis (DCNT) no Brasil 2011-2022 ^41^, que apresentam as linhas de cuidado como estratégia de intersetorialidade e focam suas ações em práticas individualizadas.

No documento *Perspectivas e Desafios no Cuidado às Pessoas com Obesidade no SUS: Resultados do Laboratório de Inovação no Manejo da Obesidade nas Redes de Atenção à Saúde*^39^, são discutidas a facilidade teórica de abordar a obesidade de forma interdisciplinar e a dificuldade de efetivar essa narrativa para além dos determinantes biológicos. A *Estratégia Intersetorial de Prevenção e Controle da Obesidade: Recomendação para Estados e Municípios*^35^ trata-se de uma narrativa intersetorial, no entanto, nas ações, não há estratégias para interação entre os diversos setores no cuidado das pessoas com obesidade. Nesse caso, o documento inclui ações multissetoriais, e não intersetoriais.

Documentos que ampliam a discussão do cuidado intersetorial têm narrativas baseadas nos DSS da obesidade e na segurança alimentar e nutricional, a exemplo da série técnica *Perspectivas e Desafios no Cuidado às Pessoas com Obesidade no SUS: Resultados do Laboratório de Inovação no Manejo da Obesidade nas Redes de Atenção à Saúde*^39^ e da PNSAN ^40^. A primeira contextualiza as dimensões da obesidade, já a segunda aborda a segurança alimentar e nutricional e a garantia do direito humano à alimentação adequada (DHAA), considerando os sistemas alimentares. Embora descrito que ações individuais são menos efetivas do que as realizadas em coletivo, a exemplo do *Caderno de Atenção Básica nº 35*^36^, de forma geral, as ações continuam pautadas em intervenções individualizadas, focadas nas escolhas alimentares e na prática de exercício físico.

Tais representações da narrativa dominante e da análise crítica sobre as ações intersetoriais, muito descritas, mas pouco operacionalizadas nos documentos, apresentam caminhos para o entendimento dos efeitos no cuidado das pessoas com obesidade, mas reduzidas possibilidades de aprofundamento e construção de afirmações neste estudo. Na análise, a discussão sobre a corresponsabilização do cuidado mostrou-se como uma lacuna.

### Obesidade e interseccionalidade: onde estão os silêncios?

Gênero/sexo, raça/cor e classe social surgem de forma coadjuvante e desmembrada nos documentos. Geralmente, são descritos nas apresentações e introduções como marcadores sociais importantes que têm relação com a obesidade, mas tal relação é pouco explorada e/ou incorporada nas estratégias e ações. A PNAN ^5^ cita que as desigualdades de renda e raça refletem nos indicadores de saúde e nutrição, exemplificando que mulheres negras pobres apresentam maiores percentuais de DCNT quando comparadas às mulheres brancas com maior renda, mas não incorpora essa disparidade nas diretrizes propostas e ações equitativas. Para além do conceito e da parte introdutória, a raça/cor não teve presença nos documentos, tampouco o racismo foi abordado como potencializador de vulnerabilidades para o surgimento e/ou agravamento da obesidade.

Já o marcador social gênero/sexo surgiu a partir de um recorte pontual, atrelado ao sistema reprodutor, como no *Caderno de Atenção Básica nº 38*^37^ e no documento *Perspectivas e Desafios no Cuidado às Pessoas com Obesidade no SUS: Resultados do Laboratório de Inovação no Manejo da Obesidade nas Redes de Atenção à Saúde*^39^, em que o ganho de peso excessivo na gestação e a paridade foram descritos como fatores relacionados à prevalência de obesidade em mulheres. Já a PNAISM ^38^ sugere que a prevenção da obesidade é importante para reduzir complicações no climatério. Foi observado que os documentos consideraram apenas o contexto de mulheres cisgênero.

A classe social foi apresentada com foco na renda e relacionada com as possibilidades de aquisição e consumo de alimentos - outras intersecções não foram observadas. Não houve, portanto, a problematização sobre renda e classe social relacionada a outros marcadores sociais, como gênero/sexo e raça/cor.

A análise crítica dos documentos identificou a interseccionalidade como um silêncio nas políticas públicas de saúde direcionadas ao cuidado da obesidade. Não foi identificada tal abordagem teórico-metodológica nos documentos analisados. Os marcadores sociais são apresentados nas políticas públicas de saúde de forma fragmentada e descontextualizados da sua intersecção com a obesidade. O gênero/sexo se relacionou aos ciclos reprodutivos de mulheres cisgênero e sua maior vulnerabilidade ao surgimento da obesidade. A classe social e a renda foram apenas determinantes para aquisição e consumo de alimentos. Considerando a raça/cor, não foi encontrado documento que contemplasse o marcador social no cuidado da obesidade.

## Discussão

Os silêncios identificados no estudo evidenciam desafios para as políticas públicas de saúde relacionadas ao cuidado da obesidade, atrelados à necessidade de inserção da interseccionalidade na construção de estratégias de promoção à saúde e prevenção da obesidade. A narrativa dominante apresentada neste artigo coaduna com outros estudos ^28^^,^^43^ que também discutiram a importância de problematizar narrativas que simplificam a complexidade da obesidade ^4^^,^^15^^,^^44^^,^^45^^,^^46^.

O estudo de Salas et al. ^28^, realizado no Canadá, que analisou políticas públicas de saúde no cuidado da obesidade utilizando a abordagem WPR, identificou narrativa dominante semelhante ao presente estudo. Os autores reforçaram a influência e a importância da narrativa dominante na obesidade, porém problematizaram que tal narrativa simplista apresenta lacunas, pois movimentar o corpo e comer de forma saudável não são ações suficientes para superar uma gama de problemas sociais atrelados ao surgimento ou agravamento da obesidade. Tal concepção corrobora os resultados descritos e a necessidade de considerar os marcadores sociais e os DSS na obesidade. Os achados de Silva & Cabrini ^11^ reafirmam as inter-relações dos sentidos afetivos, sociais, culturais e econômicos na interação do consumo de alimentos e dos corpos, ampliando, assim, a compreensão da obesidade para além do contexto biológico.

A partir do entendimento do processo saúde-doença, mediado pelos DSS, é possível compreender que o consumo de alimentos e a prática de atividade física são condicionados a uma série de determinantes, e não se configuram como o problema em si ^47^. Os meios adotados para satisfazer as necessidades, as escolhas alimentares e a adoção de um estilo de vida adequado e saudável não dependem apenas da vontade individual, mas também do contexto social, cultural, político, econômico e ambiental a que as pessoas estão submetidas ^29^^,^^46^^,^^48^.

Estudo que analisou as diretrizes brasileiras de obesidade apresentou que, mesmo existindo a narrativa de que as causas da obesidade são multifatoriais, as recomendações ainda se concentram prioritariamente nos aspectos biológicos e no cuidado em ações individualizadas focadas essencialmente no balanço energético ^46^. Resultados semelhantes também foram encontrados no estudo de Burlandy et al. ^47^, o que reforça a existência de um paradoxo, pois ao mesmo tempo que a obesidade é descrita como multifatorial, as intervenções propostas abarcam prioritariamente apenas um dos fatores: o biológico.

Caterson et al. ^49^ apresentaram em seu estudo que 81% dos participantes que viviam com obesidade acreditavam que a responsabilidade pela sua condição era apenas individual. Já estudo realizado por O’Keeffe et al. ^50^, com profissionais de saúde de 77 países e população geral maior de 18 anos de quatro países, identificou que ambos os públicos concordaram com a afirmação de que a obesidade pode ser prevenida unicamente com o compromisso dos indivíduos com um estilo de vida saudável. Os participantes que entendiam a obesidade como resultado de escolhas individuais apresentaram, no mesmo estudo, maiores escores de estigma baseado no peso e percebiam menor necessidade de priorização de financiamentos em pesquisas sobre obesidade.

À medida que a narrativa dominante atribui como soluções para os problemas representados apenas ações de controle individual, há reforço da culpabilização, estigmatização, preconceito e discriminação das pessoas com obesidade ^28^^,^^44^^,^^46^^,^^51^. Essa narrativa é produzida em um contexto neoliberal capitalista, que reforça e aquece o mercado produtivo com a criação de produtos e necessidades de consumo individuais em detrimento de ações coletivas para o cuidado das pessoas com obesidade ^4^^,^^43^.

Quanto às características de gênero/sexo, raça/cor e classe social de pessoas com obesidade, o estudo de Rubino et al. ^44^ discutiu a maior vulnerabilidade das mulheres ao estigma e à discriminação em relação ao peso, o que repercutiu na ampliação das desigualdades de educação e renda. Já Tyrrell et al. ^52^ sugeriram associação entre índice de massa corporal elevado e níveis socioeconômicos mais baixos, especialmente em mulheres. Por outro lado, a relação entre rendimentos e marcadores sociais de raça/cor e gênero/sexo demonstrou que mulheres negras têm menores rendimentos quando comparadas com todos os demais grupos, incluindo pessoas brancas, independentemente do sexo. A ausência de discussão sobre raça/cor, apresentada como um silêncio, pode ser interpretada como uma das expressões do racismo estrutural que dificulta a inserção da temática racial de forma transversal nos documentos e as discussões dos cuidados em saúde em diversos segmentos - entre eles, a obesidade -, como identificado neste estudo e já descrito por Oraka et al. ^12^.

Esses achados destacam que as intersecções atuam de forma ampla, potencializando iniquidades sociais e opressões interseccionais, e não estão ligadas apenas a questões biológicas, como as encontradas na narrativa dominante deste estudo. A interseccionalidade é uma abordagem teórico-metodológica ^17^^,^^18^^,^^19^^,^^20^^,^^21^^,^^22^^,^^23^ fundamental para o debate e para a construção das políticas públicas de saúde. Sua inserção na discussão contribui para a compreensão das iniquidades provenientes de marcadores sociais e necessitam ser contempladas como parte das soluções propostas nas políticas públicas de saúde. É preciso considerar que as opressões relacionadas aos marcadores sociais discutidos no estudo ampliam iniquidades em saúde e tornam as pessoas que as experimentam mais vulneráveis ao surgimento e agravamento da obesidade ^21^^,^^22^^,^^26^^,^^53^^,^^54^.

A intersetorialidade é uma ferramenta de potencialização da resolutividade do cuidado em saúde às pessoas com obesidade, a partir de um modelo assistencial integral e interprofissional ^43^^,^^47^^,^^55^, mas as dificuldades apresentadas na concretização de ações intersetoriais podem estar relacionadas à normatização de um cuidado em saúde biologicista, que opera a partir da queixa-conduta, bem como à dificuldade de avançar para um modelo baseado na determinação social da saúde ^47^^,^^56^. As discussões que possibilitaram compreensões ampliadas acerca da obesidade estavam presentes nos documentos que a consideravam como um problema social e que estavam centrados na segurança alimentar e nutricional e na garantia do DHAA. Isso demonstra caminhos possíveis para a compreensão ampliada das causas e das estratégias de cuidado das pessoas com obesidade nas políticas públicas de saúde, adotando a segurança alimentar e nutricional e o DHAA como fios condutores, além de incorporar uma abordagem interseccional, como a proposta no estudo que agregue a multidimensionalidade da alimentação e nutrição e dos DSS, associada ao seu caráter biológico.

A participação e o controle social nas políticas públicas de saúde e na gestão do cuidado em saúde são primordiais, especificamente das pessoas que convivem com a obesidade e têm interconexões entre os marcadores sociais de gênero/sexo, raça/cor e classe social. Tal participação ativa precisa ser um dos pilares para ampliação do olhar puramente teórico sobre os problemas representados e para inclusão do saber mediado pelas vivências. Portanto, análises críticas, como a realizada neste estudo, devem compor os processos de elaboração, implementação e avaliação de políticas públicas de saúde, na intenção de superar a perpetuação de narrativas dominantes que desconsideram marcadores sociais diretamente relacionados à obesidade.

O estudo apresenta como limitação o não aprofundamento teórico-conceitual dos sistemas de opressão concebidos a partir dos marcadores sociais apontados, expressos pelo sexismo, racismo e capitalismo, sendo de extrema relevância contemplar esses aspectos em estudos futuros. São importantes também a inclusão e a problematização de marcadores sociais de identidade de gênero, uma vez que nos documentos analisados foram consideradas apenas ações com pessoas cisgênero. Estudos que contemplem, para além de análises documentais, o contexto assistencial também se fazem necessários, para que seja possível aprofundamento dos efeitos da narrativa dominante no cuidado das pessoas com obesidade.

A interlocução entre a abordagem metodológica WPR e a perspectiva teórico-metodológica da interseccionalidade na análise crítica de narrativas das políticas públicas de saúde se configura como uma contribuição para as discussões no âmbito da elaboração e análise de políticas públicas de saúde voltadas ao cuidado de pessoas com obesidade para ampliação das narrativas, ultrapassando o domínio biológico. A utilização da WPR para análise de políticas públicas de saúde com a temática da obesidade e a incorporação da interseccionalidade ainda são incipientes na realidade brasileira, apesar do uso em outros países ^28^^,^^56^, mas ambas representam caminhos analíticos potentes.

Não foram encontrados estudos brasileiros que discutam a obesidade a partir de uma abordagem interseccional, considerando os marcadores sociais aqui apresentados. Tais achados podem contribuir para a construção de novos caminhos analíticos que superem a narrativa dominante simplista sobre obesidade nas políticas públicas de saúde, associados ao entendimento da necessidade de promover um cuidado integral, universal e equitativo às pessoas com obesidade a partir da interseccionalidade.

## Conclusão

O balanço energético positivo é a narrativa dominante das causas da obesidade, o que cria lacunas e repercute no cuidado das pessoas com obesidade, ao mesmo tempo que reduz a multidimensionalidade da obesidade a aspectos biológicos, desconsiderando as intersecções dos marcadores sociais e dos DSS. O silêncio da interseccionalidade nas políticas públicas de saúde que se relacionam ao cuidado na obesidade impacta negativamente a garantia de um cuidado integral, universal e equitativo. Contribui, ainda, para manutenção de uma narrativa simplista e individualizada sobre um fenômeno complexo e coletivo, fortalecendo compreensões estigmatizantes, preconceituosas e discriminatórias sobre pessoas com obesidade.

Este estudo não teve como objetivo deslegitimar a narrativa das políticas públicas de saúde, tampouco reduzir a importância dos aspectos biológicos como parte do cuidado da obesidade, mas sim reforçar a partir da interseccionalidade a necessidade de ampliar tal compreensão para além da narrativa dominante apresentada. Os silêncios encontrados no estudo destacam a importância de inserção da interseccionalidade na construção, implementação e avaliação das políticas públicas de saúde para o cuidado das pessoas com obesidade. É preciso contemplar os marcadores sociais de gênero/sexo, raça/cor e classe social e os impactos que os sistemas de opressões existentes exercem sobre os indivíduos e coletividades no surgimento e agravo da obesidade, extrapolando, assim, a narrativa dominante. Logo, a análise crítica de políticas públicas de saúde realizada no estudo, a partir da interseccionalidade, pode contribuir para a superação das lacunas, visando o cuidado das pessoas com obesidade.
